# The relationship of serum lipid profiles and obesity with the severity of carpal tunnel syndrome

**DOI:** 10.11604/pamj.2021.39.90.27234

**Published:** 2021-06-01

**Authors:** Athena Sharifi Razavi, Narges Karimi, Fatemeh Bashiri

**Affiliations:** 1 Department of Neurology, Clinical Research Development Unit of Bou Ali Sina Hospital, Mazandaran University of Medical Sciences, Sari, Iran,; 2 Department of Neurology, Immunogenetics Research Center, Clinical Research Development Unit of Bou Ali Sina Hospital, Mazandaran University of Medical Sciences, Sari, Iran,; 3 School of Medicine, Mazandaran University of Medical Sciences, Sari, Iran

**Keywords:** Serum lipids, obesity, carpal tunnel syndrome, electrophysiology

## Abstract

**Introduction:**

Carpal tunnel syndrome (CTS) is a prominent compressive neuropathy. There are a number of risk factors for creating CTS but the effect of these factors on the severity of CTS is unclear. In this study, we aimed to assess the correlation of serum lipid profile and obesity with the severity of CTS.

**Methods:**

this cross-sectional study was conducted on 118 patients with idiopathic CTS. Blood samples were obtained for determining the serum levels of total cholesterol (TC), triglyceride (TG), low-density lipoprotein (LDL-C), and high-density lipoprotein (HDL-C) after 12 hours of overnight fasting. The participants were then divided into two groups of normal and abnormal serum lipids. Body mass index ≥ 30 kg/m^2^ was considered as obesity. The severity of CTS was determined based on the electrophysiological results and Boston CTS Questionnaire (BCTSQ) that evaluates symptoms severity (SSS) and functional status (FSS) of patients.

**Results:**

out of 118 participants, 108 patients performed lipid profile test that 41.17%, 50.42%, 25.21%, and 20.16% of them had TC ≥ 200, TG ≥ 150, LDL-C ≥ 130, and HDL-C < 60 milligrams per deciliter (mg/dl), respectively. The mean scores of SSS in patients with dyslipidemia including the high level of TC, TG, LDL-C, and low level of LDL-C were 34.59±7.86, 34.05±8.73, 34.93±8.21, and 33.48±7.56, respectively. There was no significant association between lipid profile and the symptom severity scale of CTS (p-value > 0.05). The mean BMI of participants was 31.35±5.35 kg/m^2^, and 58.5% of them had a BMI ≥ 30 kg/m^2^. The mean score of SSS and FSS was 33.18±8.24 and 24.43±7.12 in obese patients (BMI ≥ 30 kg/m^2^), and was 34.06±7.85 and 23.06±7.67 in patients with BMI < 30 kg/m^2^. We found no significant association between obesity with the SSS and FSS (p-value = 0.53 and 0.32, respectively). In terms of the relationship between electrophysiological grading with obesity, 44 (63.8%) of patients with BMI ≥ 30 kg/m^2^ and 22 (45.8%) patients with BMI < 30 kg/m^2^ had severe to extreme severe CTS. There was no significant association between obesity and the severity of CTS (p-value = 0.054).

**Conclusion:**

the results of this study did not demonstrate an association between serum lipid profile and obesity with the severity of carpal tunnel syndrome. The findings of this study may not be extrapolated to other populations. Further studies with more samples are needed to investigate this association.

## Introduction

Carpal tunnel syndrome (CTS) is the most common focal mononeuropathy, associated with local compression of the median nerve inside the carpal tunnel at the wrist [1]. The symptoms of CTS include pain, paresthesia, and tingling or numbness of one or bilateral hands. Motor and functional impairment of the hands can happen in more severe situations [2]. The prevalence of this disorder varies in different populations, ranging from approximately 1.82% to 6% in the general adult population [3,4]. CTS can occur at any age, but its peak incidence is between 40 and 50 years. Women are also 10 times more prone to this disease than men [5,6]. Although the etiology of CTS is unknown, there are a number of risk factors, including diabetes mellitus, thyroid disease, wrist fracture, rheumatoid arthritis, amyloidosis, acromegaly, and also age and female gender [7,8]. Previous studies have indicated a relationship between high body mass index (BMI) and CTS. Evidence also suggests that BMI plays a crucial role in the development of CTS [7,9-11]. According to some studies, BMI > 30 kg/m^2^ augments fat deposition in the carpal canal and causes hydrostatic pressure over the median nerve within the carpal tunnel in the wrist [11-13].

In recent years, researchers have shown a link between CTS and metabolic syndromes. Previous studies reported that CTS is more severe in patients with metabolic syndromes, such as hypercholesterolemia, high serum low-density lipoprotein (LDL), and obesity, compared to those without such syndromes [14-17]. Hyperlipidemia is a disorder, characterized by an increase in total cholesterol (TC), triglyceride (TG), and LDL-cholesterol (LDL-C), besides a decrease in high-density lipoprotein-cholesterol (HDL-C) in the blood stream, according to the National Cholesterol Education Program guidelines (NCEP) [18-20].

Hypercholesterolemia, especially an increase in LDL-C, contributes to fibro genesis in various organs, especially peripheral nerves [16]. Consequently, hypercholesterolemia has been reported as a risk factor for idiopathic CTS. However, it is unclear the effect of lipid profile on the severity of CTS [16]. On the other hand, some studies described no correlation between hyperlipidemia and CTS [21,22]. Therefore, the purpose of the current study was to evaluate the correlation of serum lipid profile and BMI with the severity of CTS.

## Methods

### Study design, setting and participants

This cross-sectional study was conducted on patients diagnosed with unilateral or bilateral idiopathic CTS, who were referred to the neurodiagnostic section of a university-affiliated clinic (Bagheban clinic) in Sari city, Mazandaran Province, Iran over one year from November 2017 to December 2018. CTS was well-demarcated according to the American Academy of Neurology (AAN) criteria [23]. Recruitment, follow-up and data collection of CTS patients were completed in Baghban clinic. The inclusion criteria were as follows: pain, weakness, numbness, paresthesia, or tingling in one or both hands (median nerve distribution); adult patients (≥ 18 years of age); and electro physiologically confirmed diagnosis of CTS. On the other hand, the exclusion criteria were as follows: diagnosis of diabetes mellitus; hypothyroidism; rheumatoid arthritis; corticosteroid or hormone-replacement treatment; lupus; acromegaly; pregnancy; history of wrist fracture; surgery for CTS; polyneuropathy; cervical radiculopathy; and brachial plexopathy or thoracic outlet syndrome in the electrophysiological findings. All participants provided signed consent form to contribute in this study. This study was approved by the Institutional Research Ethics Review Board of Mazandaran University of Medical Sciences (ethic code: IR.MAZUMS.REC.1398.1027). It was extracted from a medical student thesis with the project number, 2861.

### Data sources and measurement

After detecting the clinical symptoms, all patients underwent electro diagnostic tests, i.e., electromyography-nerve conduction velocity (EMG-NCV) of the upper limbs at room temperature, using a MYOQUICK EMG apparatus (MicroMed, USA). EMG-NCV was conducted based on the guidelines of the American Association of Neuromuscular and Electrodiagnostic Medicine (AANEM) [23,24]. Both hands of each participant were surveyed, and the presence or absence of CTS was determined both clinically and electro physiologically. Clinical and electrophysiological evaluations were performed by one neurologist. After confirming CTS, the patients were assessed for weight (kg), height (m^2^), BMI (kg/m^2^), and serum lipid profile. BMI in this study was defined based on the definition and classification of the World Health Organization (WHO). Participants were divided to two groups, BMI < 30 and ≥ 30 kg/m^2^. Patients with BMI ≥ 30kg/m^2^ were considered obese. The participants were asked to complete a questionnaire, including demographic data, as well as the Boston Carpal Tunnel Questionnaire (BCTQ). BCTQ evaluates the severity of symptoms (SSS) and functional status score (FSS) of patients with CTS [25]. The SSS scale contains 11 questions, the scores of which represent five levels of SSS: 1= no symptoms; 2= mild; 3= moderate; 4= severe; and 5= very severe.

Moreover, the FSS scale includes eight items, each with five levels of difficulty: 1= no difficulty; 2= mild difficulty; 3= moderate difficulty; 4= severe difficulty; and 5= very severe difficulty. The total score of SSS and FSS are summed [25]. The severity of CTS in patients was based on BCTQ and electrophysiological (EMG-NCV) results. The EMG-NCV studies were conducted using standard techniques of supramaximal percutaneous stimulation with a surface electrode and a persistent current stimulator [26]. Parameters used to evaluate sensory median nerve included the peak latency (PL), amplitude of sensory nerve action potential (SNAP), and conduction velocity (CV). The motor median nerve was also assessed based on distal motor latency (DML), amplitude of compound muscle action potential (CMAP), and CV. In median sensory studies, PL > 3.5 ms, base-to-peak amplitude < 20.0 μV, and CV < 50 m/s were considered abnormal. In line with median motor studies, DML > 4.4 ms, base-to-peak amplitude < 4.0 mV, and NCV < 49 m/s were considered abnormal [17,27-29]. The electrophysiological results were classified into six grade (0-6), from normal to extremely severe CTS, based on bland neurophysiological grading scale [30]. Electrophysiological study was performed in bilateral hands and the most affected hand with worse electrophysiological findings was included in the grading of CTS.

### Blood collection and analysis

Blood samples were obtained for determining the serum levels of TC, TG, LDL-C, and HDL-C between 7: 00 am and 10: 00 am after 12 hours of overnight fasting. All blood analyses were performed in Bagheban university-affiliated clinic laboratory on the day of sample collection. The participants were then divided into two groups of normal and abnormal serum lipid profile, according to the National Cholesterol Education Program (NCEP) ATP III definition [19]. Serum TG concentration ≥ 150 mg/dL, HDL-C < 60 mg/dL, TC ≥ 200 mg/dL, and LDL-C ≥ 130 mg/dL were considered abnormal. The two groups were compared regarding the severity of CTS according to the BCTQ and electrophysiological findings. Electrophysiological study was performed in bilateral hands and the most affected hand with worse electrophysiological findings was included in the grading of severity and statistical analysis.

### Statistical analysis

Statistical studies were performed in IBM SPSS Version 24. Differences between the groups were examined using independent sample t-test and Chi-square test. Data were expressed as mean±SD. Independent sample t-test was carried out to compare quantitative variables, including electrophysiological parameter results and the mean of SSS and FSS between two groups of normal and high level of lipid profile. For assessing of relationship between obesity (BMI ≥ 30 kg/m^2^) and lipid profile was used Chi-square test. P-values < 0.05 were considered statistically significant.

## Results

**Figure 1:**
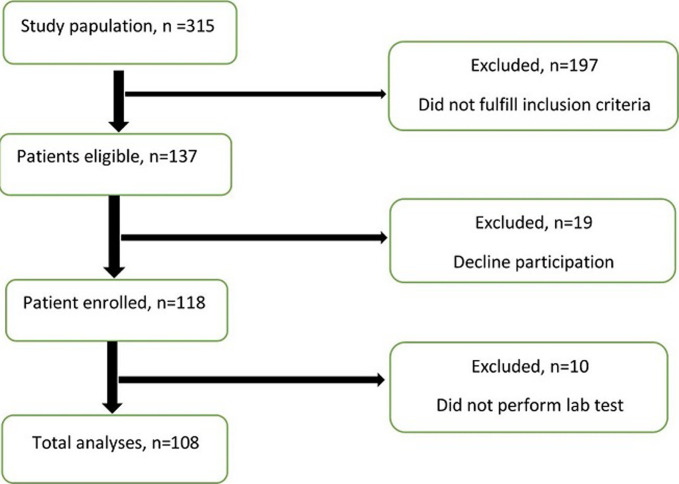
clow chart of study population according to the STROBE-recommendations; from the initially included 137 patients, 29 were excluded from further analysis because of not participating in the study and not doing profile lipid test

A total of 118 participants were enrolled in this study out of 137 samples, according to the clinical examination and electrophysiological results (Figure 1). Electrophysiological tests were performed separately for each hand. Overall, 236 electrophysiological examinations were carried out. Among patients included in the study, 100 (84.7%) were female, and 18 (15.3%) were male. Ten out of 118 patients, including eight women and two men, did not undergo lipid profile test and lipid profile test was performed in 108 participants. The mean age of the participants was 44.64±8.43 years, and the most frequent age group was 40-49 years (40.7%). The contributors had a mean BMI of 31.35±5.35 kg/m^2^, and out of 118 patients, 69 (58.5%) of them had a BMI ≥ 30 kg/m^2^. In terms of CTS symptoms in the involved hand, 49 (41.52%) patients mentioned having bilateral symptoms, while 69 (58.57%) reported unilateral symptoms (40 and 29 patients in the right and left hands, respectively). After electrophysiological examinations, it was found that 105 (89%) patients had bilateral hand involvement.

### Evaluation of serum lipid profile in patients with CTS

Serum levels of TC ≥ 200 mg/dl, LDL-C ≥ 130 mg/dl, TG ≥ 150 mg/dl, and HDL-C < 60 mg/dl were reported in 49 (41.17%), 30 (25.21%), 60 (50.42%), and 30 (20.16%) of patients, respectively. The mean serum levels of TC, TG, LDL-C, and HDL-C were found to be 201.29±38.10 mg/dL (range: 50-285 mg/dL), 185.16±101.71 mg/dL (range: 65-803 mg/dL), 116.88±30.84 mg/dL (range: 26-207 mg/dL), and 58.42±35.06 mg/dL (range: 30-280 mg/dL), respectively. In terms of the relationship between the age of patients and lipid panel, there was a significant association between the serum level of LDL-C and the age of patients. The mean age of patients with LDL-C level ≥ 130 mg/dl was 47.51±8.26 and < 130 mg/dl was 43.12±8.12 (P = 0.012). In respect of the relationship between obesity (BMI ≥ 30 kg/m^2^) and serum lipid profile, there was a significant association between obesity and only TG ≥ 150 mg/dl. Out of 62 patients with BMI ≥ 30 kg/m^2^, 40 (64.5%) patients had TG ≥ 150 mg/dl and out of 46 patients with BMI < 30 kg/m^2^, 20 (43.4%) had TG ≥ 150 mg/dl (OR = 2.36; 95%CI, 1.08-5.16; p = 0.024). An odds ratio of 2.36 means the odds of having TG ≥ 150 was 2 times higher among patients with BMI ≥ 30 kg/m^2^ than BMI < 30 kg/m^2^. Table 1 demonstrates the serum lipid profile of patients according to age, sex, involved hand, and BMI.

**Table 1:** the association of demographic findings and serum lipid profile

	Total Cholesterol (mg/dL)			Triglyceride(mg/dL)			LDL-C(mg/dL)			HDL-C (mg/dL)		
Variables (n)	Normal (59)	Abnormal (49)	P-value*	Normal (48)	Abnormal (60)	P-value*	Normal (74)	Abnormal (34)	P-value*	Normal (80)	Abnormal (28)	P-value*
**Age, mean±SD**	44.23±8.63	45.05±8.36	0.60	43.93±9.12	45.16±7.95	0.45	43.12±8.12	47.86±8.34	0.012	44.87±8.54	43.20±8.04	0.35
**Gender,n(%)**			0.33			0.19			0.25			0.10
Male	9 (56.2)	7(43.8)		5 (31.3)	11(68.7)		13(81.3)	3(18.7)		12(75)	4(25.0)	
Female	50 (54.3)	42(45.7)		43(46.7)	49(53.3)		61(67.0)	30 (33.0)		68 (74.7)	24(25.3)	
**BMI, n(%)**			0.26			0.024			0.44			0.48
< 30 kg/m^2^	23(50.0)	23 (50.0)		26 (65.2)	20 (43.5)		31(56.2)	15(32.6)		35 (76.1)	11(23.9)	
≥ 30 kg/m^2^	36 (57.1)	26 (41.9)		22 (35.5)	40 (64.5)		43 (70.5)	19 (29.5)		46 (74.2)	16(25.8)	
Bilateral CTS, n(%)	23 (48.9)	24(51.1)	0.27	19 (40.4)	28 (59.6)	0.14	34(72.3)	13(27.7)	0.35	33(70.2)	14(20.8)	0.59

* The threshold for statistical significance was p<0.05. LDL-C: low density lipoprotein cholesterol, HDL C: high density lipoprotein cholesterol.

### The relationship between demographic data and serum lipid profile with the severity of CTS according to the BCTQ

Overall, the mean scores of SSS and FSS based on the BCTQ were 33.51±8.06 (range: 14-52) and 23.86±8.35 (range: 8-40), respectively. The average scores of the SSS and FSS were compared to gender. The mean score of the SSS was 34.29±7.78 in the female group and 29.22±8.44 in the male group. Therefore, there was a significant association between SSS and gender (P-value = 0.013; 95%CI: 32.04, 34.98). Regarding FSS, the females had more difficulty with routine activities than males (24.64±7.21 vs. 19.55±6.78, p value = 0.006; 95% CI: 22.52, 25.20). The mean scores of the SSS in patients with abnormal TC, TG, LDL-C, and LDL-C were 34.59±7.86, 34.05±8.73, 34.93±8.21, and 33.48±7.56, respectively. Table 2 presents the results regarding the association of CTS severity according to the BCTQ score, lipid profile, and p-value for each. There was no significant association between the serum lipid profile and SSS or FSS of CTS patients (P > 0.05).

**Table 2:** the comparison of normal and abnormal lipid profile with the mean of symptoms severity and functional status scale according to BCTQ

Lipid profiles (Mg/dl)	Symptoms severity scale (mean±SD)	*p value	Functional status scale (mean±SD)	*p value
**Total cholesterol**		0.68		0.79
<200	33.33±7.80		23.89±7.35	
≥200	33.98±7.26		24.29±7.63	
**Triglyceride**		0.55		0.43
<150	33.14±7.02		23.50±7.67	
≥150	34.05±8.73		24.53±7.31	
**Low density LP**		0.38		0.35
<130	32.91±8.71		23.43±7.41	
≥130	34.40±8.05		24.93±6.97	
**High density LP**		0.81		0.27
<60	33.16±8.71		23.04±7.32	
≥60	33.52±6.98		24.68 ±7.51	

LP: lipoprotein; *The threshold for statistical significance was p<0.05.

### Comparison of serum lipid profile with electrophysiological parameters

Based on the relationship of serum lipid profile with the electrophysiological findings, there was no evidence that the high serum levels of TC, TG, LDL-C, and low level of HDL-C have an influence on electrophysiological parameters (t-test analysis, p-value > 0.05). Table 3 demonstrates the relationship between the mean of sensory and motor median nerve action potentials in CTS patients with a normal and abnormal serum level of lipid profile and amounts of p-value.

**Table 3:** the relationship of serum lipid profiles with electrophysiological findings

Lipid profiles (Mg/dl)	SNAP(mean±SD)	CMAP (mean±SD)				
	PL (ms)	A (µv)	CV (m/s)	DL (ms)	A (mv)	CV (m/s)
**Total cholesterol**						
<200	6.49±2.66	17.18±13.47	27.68±11.39	6.21±1.70	10.69±5.26	53.57±7.69
≥200	6.99±3.00	13.09±11.73	26.01±12.16	6.30±1.67	11.22±5.45	53.04±9.37
*p-value	0.37	0.09	0.46	0.80	0.61	0.74
**Triglyceride**						
<150	6.31±2.66	17.10 ±13.60	28.98±11.60	6.16±1.87	10.29±4.80	54.10±8.98
≥150	7.04 ±2.92	13.91±12.09	25.28±11.65	6.32±1.52	11.45±5.70	52.72±8.06
*p-value	0.18	0.19	0.10	0.63	0.26	0.39
**Low density**						
<130	6.61±2.79	16.26±13.02	27.24±11.69	6.22±1.67	10.57±5.06	53.11±7.52
≥130	6.82±2.87	13.62±12.26	26.47±11.81	6.17±1.49	11.98±5.76	53.92 ±10.46
*p-value	0.72	0.32	0.82	0.88	0.20	0.65
**High density**						
<60	6.56±2.74	15.41 ±12.81	26.24±11.25	6.32±1.37	11.00±6.98	51.10±9.20
≥60	6.72±2.84	15.49 ±12.60	27.36±11.87	6.17±1.07	11.00±4.65	53.10±8.30
*p-value	0.80	0.97	0.67	0.66	0.99	0.29

LP: lipoprotein; SNAP: sensory nerve action potentials; PL: peak latency; A: amplitude; CV: conduction velocity; CMAP: median nerve compound muscle action potential; DL: distal latency. *The threshold for statistical significance was p<0.05.

### The relationship serum lipid profile and obesity with the severity of CTS on the basis of electrodiagnostic grading

**Figure 2:**
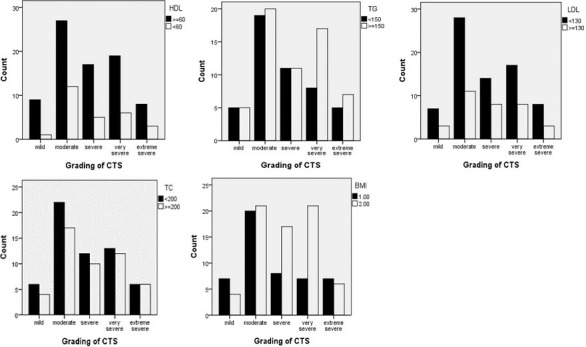
comparison of serum level of lipids with electro-diagnostic grading of CTS, [total cholesterol (TC), triglyceride (TG), high-density lipoprotein (HDL) and low-density lipoprotein (LDL)]

In terms of the CTS severity according to the electrodiagnostic grading, the prevalence of mild, moderate, severe, very severe, and extreme severe CTS was 11 (9.24%), 41 (34.45%), 25 (21%), 28 (23.52%), and 13 (10.92%), respectively. Moreover, the grading of CTS was compared with the serum lipid profile and obesity; the results are summarized in Figure 2. The chi-square statistical analysis found no significant relationship between serum level of TC, TG, LDL, and HDL with the electrodiagnostic grading of CTS (P-value = 0.97, 0.68, 0.74, 0.97, respectively). In terms of the relationship between electrophysiological grading with the obesity, out of 69 participants with BMI ≥ 30 kg/m^2^ and 48 patients with BMI < 30 kg/m^2^, 44 (63.8%) and 22 (45.8%) of patients had severe to extreme severe CTS, respectively (Figure 2). There was no significant association between obesity with the severity of CTS (p-value=0.054).

### The relationship between obesity and severity of CTS according to BCTQ

BMI ≥ 30 kg/m^2^ was reported in 69 (58.48%) of patients, including 62 (62%) females. The mean score of SSS and FSS was 33.18±8.24 and 24.43±7.12 in obese patients (BMI ≥ 30 kg/m^2^), and was 34.06±7.85 and 23.06±7.67 in patients with BMI < 30 kg/m^2^). Independent sample t-test analysis found no significant differences between obesity and severity of CTS (p-value=0.53 and 0.32 respectively).

## Discussion

This study set out with the aim of correlation the serum level of lipid profile and body mass index with the severity of CTS according to the electrophysiological findings and Boston score. In the present study, about half the participants had hypercholesterolemia and hypertriglyceridemia but there was no correlation between serum lipid profile with the severity of CTS based on Boston score. Also, no alterations were observed in electrophysiological findings among patients with normal and abnormal lipid profile. This despite the fact that some studies had shown that a correlation between serum LDL-C and severity of CTS [13-16]. Several studies reported that elevated TC and LDL are related with the increased of CTS, and high HDL is predictable as a protective factor for CTS [16]. Nakamichi *et al*. also reported that distal motor latency and sensory nerve CV were correlated with the serum level of LDL-C, but there was no significant correlation with high TG or low HDL-C [16]. Bischoff *et al*. in a case-control study, found no significant difference in the serum LDL-C between patients with CTS and healthy individuals, which is in line with our study [21]. On the other hand, Yeo *et al*. reported a positive correlation between TG level and CTS severity. However, TC, LDL-C, and HDL-C were not significantly correlated with CTS severity [22]. Yurdakula *et al*. also showed that electrophysiological findings and severity of CTS were worse in patients with metabolic syndromes [14].

In this study, the overall BMI was 31.34±5.34 kg/m^2^, and more than fifty percent of the patients had a BMI of higher than 30 kg/m^2^. The rates of being overweight and obese in the current study were higher than those reported in other studies [14]. A significant correlation was found between obesity and female gender, but there was no significant relationship between obesity and severity of clinical symptoms or electrophysiological findings. Previous studies reported that obesity is a risk factor for CTS [13,30-33] but the relationship of obesity with the severity of CTS has been disputed. Komurcu *et al*. reported that BMI is correlated with CTS severity according to electrophysiological findings [11]. Becker *et al*. also found that BMI was significantly different between the groups with mild to moderate, and severe CTS [13]. In addition, Kurt *et al*. demonstrated that high BMI increases the severity of CTS [34]. While some studies found that higher value BMI was a major contributor to CTS, it was unrelated to the severity of CTS, similar to our study [32,35]. Therefore, this result may be explained by the fact that high value BMI may be as a risk factor of CTS as result of the enlarged fatty tissue and the increased hydrostatic pressure into the carpal canal in the overweight persons but for assessing between BMI and severity of CTS, more extensive study needs to be accomplished.

The present study showed that CTS is more common in females (female-to-male ratio= 5.5: 1), which is in line with previous studies [8,11,14,36]. A significant association was found between gender and Boston score severity. The clinical symptom severity and disturbance in daily activities were more severe in females than males.

## Conclusion

The present study found no relationship between lipid profile and high BMI with the severity of CTS, either based on BCTQ or electro diagnostic findings. There are other factors that may play an important role on the severity of CTS. Further studies are needed with a larger sample size to investigate the impact of the hyperlipidemia and obesity on the severity of CTS and compare patients with the healthy populations.

### What is known about this topic

- Several risk factors contribute to the development of CTS including diabetes mellitus, thyroid disease, wrist fracture, rheumatoid arthritis, amyloidosis, obesity, and acromegaly;
- Hypercholesterolemia has been reported as a risk factor for carpal tunnel syndrome;
- It is uncertain the relationship hyperlipidemia and obesity with the severity of idiopathic CTS;

### What this study adds

- No association were found between lipid profile and obesity with the severity of CTS, either based on Boston carpal tunnel score or electrodiagnostic findings;
- Serum lipid levels and obesity are not associated with the severity of electrodiagnostic findings;
- There was a relationship between the clinical symptoms severity of CTS and female gender.
